# Connection Over Convenience: Why Communication Matters More Than Logistics in LTC Visits

**DOI:** 10.1093/geroni/igaf122.3739

**Published:** 2025-12-31

**Authors:** Nicole Praska, Caroline Collins-Pisano, Rachel Weiskittle

**Affiliations:** University of Colorado Colorado Springs, Colorado Springs, Colorado, United States; University of Colorado Colorado Springs, Colorado Springs, Colorado, United States; University of Colorado, Colorado Springs, Colorado Springs, Colorado, United States

## Abstract

Residents of long-term care (LTC) facilities often experience social isolation, even with regular visits from family and friends. Satisfaction with these visits supports relationship maintenance and residents’ emotional well-being. Both structural factors and interpersonal dynamics may shape visitor satisfaction, yet their relative contributions remain underexplored. Identifying these predictors can inform programs that foster family engagement and enhance residents’ social well-being. This cross-sectional study examined whether LTC facility characteristics (facility type, travel time, length of stay) and resident communication factors (verbal communication ability, recognition of visitor, alertness) were associated with visitor satisfaction. An online national survey was completed by 175 community-dwelling adults (M age = 49.58, SD = 17.86, range 20-82) who had visited a loved one in LTC within the past 12 months. Pearson correlations assessed associations between visitor satisfaction and key variables. No significant associations emerged between visitor satisfaction and facility characteristics (years living in facility: B = 0.05, p = .09; minutes traveled: B = 0.001, p = 0.82; independent/assisted living: B = 0.19, p = 0.46; nursing home/skilled nursing: B = 0.17, p = 0.42). Verbal communication ability was significantly positively associated with visitor satisfaction (B = 0.18, p = 0.02). Recognition of visitor and alertness were not significant predictors (recognition: B = 0.04, p = 0.61; alertness: B = –0.08, p = 0.39). Findings suggest that residents’ verbal communication may play a more critical role in shaping visitor satisfaction than structural or logistical aspects of visits.

